# Vaccine Hesitancy and Perceptions of the Community about Polio in High-Risk Areas of Karachi, Sindh, Pakistan

**DOI:** 10.3390/vaccines11010070

**Published:** 2022-12-28

**Authors:** Fayaz Hussain Abbasi, Ahmed Ali Shaikh, Jaishri Mehraj, Syed Musa Raza, Shumaila Rasool, Umar Farooq Bullo, Sandeep Mehraj, Zamir Ali Phul, Sundeep Sahitia, Asif Ali Zardari, Shoukat Ali Chandio

**Affiliations:** 1 Emergency Operations Centre for Polio Eradication and Immunization, Government of Sindh, Karachi 75510, Pakistan; 2 Bill and Melinda Gates Foundation, Islamabad 44000, Pakistan; 3 Integral Global Health Inc., Islamabad 44000, Pakistan; 4 The United Nations Children’s Fund (UNICEF), Islamabad 44050, Pakistan; 5 National Stop Transmission of Polio (N-STOP) Program, Karachi 75510, Pakistan; 6 World Health Organization (WHO), Islamabad 45500, Pakistan

**Keywords:** vaccination, vaccine coverage, vaccine acceptance, vaccine hesitancy, Pakistan

## Abstract

The study aimed to determine the reasons for polio vaccine hesitancy among parents of persistently missed children (PMCs) in the high-risk areas of Karachi, Pakistan. A cross-sectional survey of parents of PMCs was conducted in April 2019 in 34 high-risk union councils of Karachi. PMCs were randomly selected from the polio program database, and further information was collected on a questionnaire by trained staff using face-to-face interviews with parents of PMCs. A total of 325 participants were included in the study. Among refusals, 112 (37.3%) had no trust in vaccine quality, followed by 45 (15.0%) who were afraid of side effects, 42 (14.0%) whose elders did not allow polio vaccination, 39 (13.0%) who refused due to the influence of negative social media videos, and 20 (6.7%) who had no trust in polio teams. We concluded that misconception is still a big challenge, and the program needs to strive for community acceptance. Low levels of trust in vaccines and teams as well as fear of OPV side effects were among the main reason for vaccine hesitancy. The participant communities recommended involving famous medical doctors, religious influencers, and TV or sports stars to enhance knowledge and acceptance of polio vaccination.

## 1. Introduction

Polio is an enteric virus that causes permanent flaccid paralysis [1]. In 1988, after considering the operational ease of a cost-effective vaccine, the World Health Assembly agreed to eradicate polio from the world within 12 years [2]. As a result, it was assumed that the world would commemorate the century with a polio-free planet. It was also found that by the year 2000, the target year for eradication, the world had achieved a 99% reduction in polio cases [3]. Even 20 years after the century, certain nations in the world failed to make any headway in stopping the spread of the poliovirus. Currently, Pakistan and Afghanistan are the last two nations in the world that have indigenous poliovirus and are known as the last two main reservoirs of wild poliovirus [4].

It was thought that Pakistan was close to achieving the objective of polio eradication in the years to come because the country reported the fewest polio cases in history between the years 2017 and 2018 [5]. However, on the second day of the national campaign to eradicate polio, a mob gathered in Peshawar, Khyber Pakhtunkhwa (KPK) Province, Pakistan, in April 2019. They claimed that the polio vaccine had resulted in an unintended incident in the children who received OPV [6]. The event received tremendous coverage on electronic and social media, which put an end to the nation’s ongoing anti-polio campaign. The event has had a significant negative impact on the current campaign, and it has also made future campaigns difficult to carry out. After April 2019, the program was placed on hold, and the experts assembled to consider the possibility that any occurrence may be enough to derail the previous success. Moreover, a social media campaign was initiated to reinforce the nation’s negative perspective of polio, which was headed by the Prime Minister’s focal person for polio eradication at that time. The campaign was given a four-month break to reassess the communication and operational modes. The level of community trust, however, remained consistently low. Following this, there was a significant increase in polio cases throughout the country, bringing the total cases to 146. In March 2020, the program was hit by the COVID-19 pandemic, and the polio eradication activities were affected due to the restriction of movement. A slight increase in administrative coverage was observed from December 2020 to January 2021. The country has always struggled in finding the optimal coverage to disrupt the circulation of the virus. The claimed coverage did not meet the disruption threshold for the core reservoir area. The primary reason for the low coverage was the acceptance of the polio vaccine by parents. It is a universal fact that the success of community programs has resulted in fewer cases of the disease; consequently, it lessened the perception of the community about the disease [7,8]. The denials occurred in clusters in the country’s major cities and later in the country’s rural areas.

Misconceptions and fear regarding the polio vaccine led to low immunization rates in the national anti-polio campaign. In addition, incidents like that in Peshawar further set back acceptance of the oral polio vaccine (OPV) [6]. Khyber Pakhtunkhwa Province, which contains Pashtun and tribal villages, became the main site of sectarian resistance to the polio vaccination. In the past, the acceptance of polio vaccination was high among the native populations of Karachi, which is among the core reservoir areas of poliovirus. However, after the Peshawar incident in 2019, the people of Karachi began to refuse the polio vaccination, and Karachi has the highest number of OPV refusals in the country.

The high-risk population in Karachi is mainly the Pashto-speaking population (Pushtun), which migrated from Peshawar and other parts of KPK province to Karachi for livelihood. Pushtun individuals living in Karachi still have very strong relationships with their relatives back in KPK. As the Peshawar incident took place, the campaign was not only halted in Peshawar but across Pakistan. Families in Karachi were told not to administer drops to their children by their ancestors and leaders from Peshawar. Therefore, the incident led to a large record of refusals in the subsequent campaign.

Karachi, located in Sindh province, is the largest metropolitan area in the country [9]. Karachi division is administratively divided into 6 districts and 192 union councils (UCs). A UC is the smallest administrative unit within a district. Out of these 192 UCs, there are 34 high-risk UCs [10]. The Polio Eradication Initiative (PEI) has divided the program into four tier classifications at the district level across the country, with a tier one district being at the highest risk and a tier four district being at the lowest risk [11]. Within districts, the UCs are also classified further on a similar basis. Out of 192 union councils in Karachi, 34 UCs are documented as high-risk UCs (HRUCs) and 12 as super-high-risk UCs (SHRUCs) based on the following criteria: It is a polio reservoir (persistent poliovirus circulation detected as either polio cases or poliovirus in the sewage (environmental sites)). The population is underserved and dense with poor health structure, clustering of households refusing vaccinations, or settlements of mobile/migratory populations. In addition, the immunization level is low in the area for both polio and routine. HRUCs were normally the neighboring/adjacent UCs to SHRUCs with poor campaign indicators such as a high percentage of missed children, and UCs which failed in lot quality assurance sampling (LQAS) due to operational gaps reported in previous multiple campaigns [11].

Low coverage and high refusal rates in the country lead the program to initiate new strategies and innovative approaches to address vaccination in the community. The Peshawar incident changed the program direction. The program leaders felt a dire need for an assessment of community perception and a subsequent revision of the approaches. It was vital to study the new hesitancy and reluctance in the community. To date, the challenge of persistently missed children (PMCs) remains a big worry for the polio eradication program as the program keeps missing the same children over time and keeps vaccinating already vaccinated children. The paradigm shifts in the program where the focus was shifted from vaccinated to missed children need further acceleration toward PMCs as these are some of the most vulnerable children. Therefore, this study was conducted to identify reasons for persistently missed children (PMCs) in 34 high-risk UCs of Karachi, to explore awareness of polio disease in the community, and to determine a way forward.

## 2. Materials and Methods

The study was conducted in Karachi city of Sindh province in Pakistan. Karachi division has a population of 14,910,352, making it the world’s 6th most populous cosmopolitan area [12]. The study sample was taken from 34 HRUCs and SHRUCs. The 34 HRUCs are in 15 towns out of a total of 18 towns in the 6 districts of the Karachi division. The 34 HRUCs are spread within 35 km^2^, which is 0.09% of the total 3780 km^2^ area of Karachi. The total population of these 34 HRUCs is 4,281,755, and the number of children below five years of age is 727,899. The 34 HRUCs are dominated by a priority-1 population and have remained the main poliovirus reservoirs and amplifiers. The 34 HRUCs also have demographic and epidemiological links with KPK/Tribal Districts, Quetta Block, and Afghanistan [11]. 

A cross-sectional survey of parents of recorded persistently missed children (PMCs) was conducted in April 2019 during a polio campaign/national immunization day (NIDs). A PMC is a child who is missed for OPV for 3 consecutive rounds. For this survey, children who were recorded as persistently missed for the 3 consecutive rounds within the last 12 campaigns in 34 HRUCs of Karachi were selected. Parents of children under five years of age, who live in the HRUCs, and who refused the OPV for more than two campaigns were included in the survey. Those parents who refused to give interviews and children who were guests of the selected households were excluded. The Emergency Operations Center (EOC) of Sindh province established by the Government of Pakistan is responsible for the overall coordination and implementation of polio vaccination campaigns in Sindh province with strong support from PEI partners. The database of polio vaccination campaigns, coverage, and still-missed children (SMCs) and PMCs is also maintained at EOC Sindh. As per the EOC Sindh polio program database, a total of 30,589 PMCs were reported from 192 UCs of Karachi and 8581 PMCs were recorded in 34 HRUCs during the March 2019 campaign. 

A questionnaire was developed and pre-tested at the provincial EOC in Karachi, Sindh, Pakistan. The questionnaire collected information on health problems and health-seeking behavior, uncovered reasons for persistently missed polio vaccination, and identified how communication about polio can be improved. The questions were developed after the literature review. The questions were drafted in English language and were translated into the local language Urdu. The questionnaire was tested for reliability using Cronbach’s alpha coefficient among the 10 selected individuals with the proportionate sample as per the designed sample. We yielded a Cronbach’s alpha coefficient of 0.71; therefore, the questionnaire was consented to by all authors for the data collection (details in Supplementary File).

Before the start of the campaign, all frontline workers (FLWs), including community health workers (CHWs) and area supervisors, were trained for the administration of the polio vaccine and interpersonal communication (IPC) by the master trainers (MTs). Twenty-three MTs were trained to conduct interviews with the parents of recorded PMCs. The training was divided into two halves. The first half familiarized MTs with each question listed in the questionnaire and trained them on how to present and introduce themselves at each household. The second half of the training involved mock sessions where MTs were divided into teams of two: one member acted as a household parent, and the other acted as the interviewer. Out of 23 MTs, 21 MTs qualified for the training. The trained MTs were divided into three categories and were assigned UCs accordingly. The three MTs were assigned three UCs. The 3 best MTs were selected on their performance indicators such as coordination, training plan, field support, and weekly reporting. Soft skill evaluation attributes were communication and interpersonal skills, leadership, organized approach, professional conduct, and result orientation (details provided in the Supplementary File). In addition, 7 MTs were assigned 2 UCs each and 11 MTs were assigned 1 UC each.

In total 10 questionnaires were completed in each UC. Each MT was given a pool of 15 households to conduct at least 10 questionnaires in each high-risk UC. One questionnaire in UC Baloch Goth was unable to be completed due to the assigned MT being bitten by a dog. Incomplete questionaries were not included, resulting in 325 completed questionnaires. The questionnaire data were then compiled by data support officers (DSOs). The data entry exercise was completed in three days. Data entry was performed twice and matched for validation. 

For sample size calculation, the total number of 8581 PMCs recorded in the March 2019 campaign in 34 HRUCs was taken as the population size. We used the estimated proportion of 50% frequency of outcome (knowledge factor) in the population, a confidence interval of 95%, and a margin of error of 5%. A minimum sample size of 368 was obtained. We used online open epi software for sample size calculation [13]. To gain insight from all 34 HRUCs and to address the possible lack of response or missing information, we randomly selected 15 PMCs per UC (a total 510 PMCs) from the polio database. We advised master trainers to obtain information from at least 10 households from each UC. Information was gathered from selected participants during NIDs in April 2019. Due to extensive media coverage of the KPK incident of April 2019, community resistance increased, and we were able to obtain responses only from 325 (88.3%) of the required 368 sample size. 

The PMC database belongs to the polio program of the EOC Sindh. The study was conducted by the technical team of EOC Sindh who has access to the PMC database. The data were obtained from the database for the study purpose after approval from the head of the EOC Sindh. The approval was obtained from the Ethics Committee of The Directorate General Health Services, Health Department, Government of Sindh. The data were entered into the Microsoft Excel program and analyzed using Statistical Package for Social Sciences (SPSS) version 24. 

## 3. Results

A total of 325 participants (parents of PMCs) were interviewed during the campaign days. One hundred forty (43%) were from district West. The number of reported children in each household ranged from 1 to 10, also including children from Pashto-speaking joint/multi-family households. One child was reported in 59 (18.1%) households, two children in 89 (27.4%) households, three children in 59 (18.2%) households, four children in 42 (12.9%) households, and five or more children were reported in 76 (23.4%) households. One hundred eighty-four (56.6%) male children were persistently recorded missed during the last 12 SIAs. Two hundred six (33%) respiratory tract infections were found to be the most common cause of childhood health problems in the area. A total of 219 (67.4%) parents visited a healthcare facility within the last 3 months for the treatment of their child. A total of 264 (81.2%) respondents preferred private healthcare facilities for the treatment of childhood health problems. Among participants, 161 (49.6%) reported that their children completed the routine immunization course, and 105 (32.3%) also had EPI vaccination cards. Characteristics of study participants in 34 high-risk union councils of Karachi division are shown in Table 1.

Supplementary Table S1 shows the union council-wise distribution of study participants in 34 high-risk union councils of Karachi division, Sindh, Pakistan.

A total of 322 (99.1%) participants allowed the polio team to visit their house during each campaign, irrespective of polio vaccine acceptance by the families. Among these, 194 (59.7%) reported that polio teams visited 11–24 times in a year. When asked about staff, 15 (4.6%) gave a rating of 0 (0 being very bad and 10 being very good) to the behavior of polio teams, whereas 139 (43.3) were fully satisfied with the behavior of the polio teams and gave a score of 10 (very good). One hundred thirty-eight (42.5%) parents gave scores of 0 (0 being not important and 10 being highly important) when asked about the importance of the polio vaccine, 132 (40.7%) gave scores of 1 to 9, and 46 (14.2%) considered polio vaccine as highly important (full score 10) for the health of their children. The fact that polio can cause paralysis was reported by 74 (22.8%), 130 (40.0%) reported that it was a type of disease, and 116 (35.7%) reported not knowing about polio. Two hundred eighteen (67.5%) were of opinion that polio team visits are for improving the health of their children, and 115 (35.6%) had the opinion that teams are visiting for their jobs/as per the requirement of the government. Among those who refused the polio vaccine, 112 (37.3%) had no trust in vaccine quality, followed by 45 (15.0%) who were afraid of side effects, 42 (14.0%) who responded that their elders did not allow polio vaccination, 39 (13.0%) who responded that negative social media videos were the main factor of their polio vaccination refusal, and 20 (6.7%) who had no trust in polio teams (Table 2). Among refusal cases, a total of 242 (74.5%) responded that someone from the polio program came to convince the refusing parents to get their child vaccinated with OPV. Among these, 136 (45.5%) did not vaccinate their children and have not considered requests from any of the higher officials of the polio program. The source of polio campaign information was the CHW/polio team for 260 respondents (57.1%), followed by TV for 68 (14.9%) and banners/pamphlets for 49 (10.8%).

**Figure 1:**
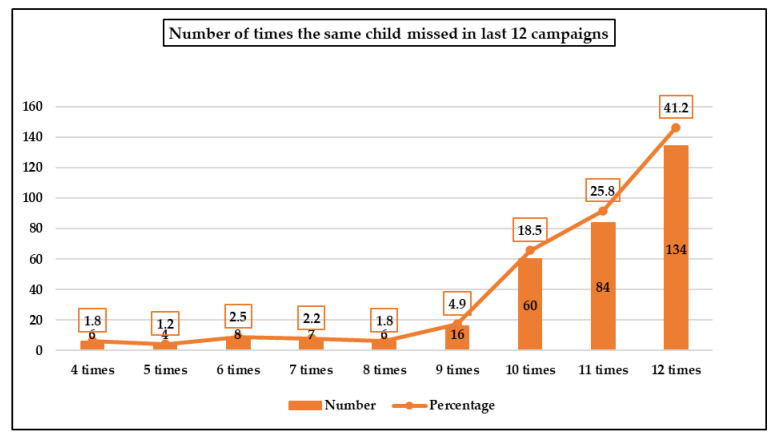
The number of times the same child was recorded as missing polio vaccination during the last 12 campaigns from Jan 2018 to March 2019 SIAs in 34 high-risk union councils of Karachi division, Sindh, Pakistan.

The analysis of the polio teams’ database indicated that the number of times that children were recorded as missed in each household ranged from 4 to 12 during campaign days in the last 12 SIAs. Only 6 (1.8%) children were recorded missed four times in the last 12 SIAs and 134 (41.2%) were recorded missed in every campaign in the last 12 SIAs (Figure 1).

Supplementary Table S2 illustrates the changing pattern in reasons for missed children in 34 high-risk union councils of Karachi division, Sindh, Pakistan. It shows a snapshot that captures a switch in reasons for missed children from one reason for refusal to another reason for refusal and a description of the switch from reasons for refusal to not available and from not available to refusal in different campaigns from Jan 2018 to March 2019 SIAs.

During every visit, polio teams also recorded reasons for the missed child as reported by the parents. In 62 (19.1%) cases, reasons for missed children remained the same across 12 SIAs, whereas in 263 (89.9%) cases, the reason for the missed child was changed by the parent of the child. In the 62 cases where the same reason persists in 12 SIAs, 9 (2.8%) were direct refusal, 38 (11.7%) refused due to some misconceptions, 1 (0.3%) refused due to religious reasons, 2 refused for demanding other services (0.6%), 9 (2.8%) refused due to some sickness of the child, and 3 (0.9%) always reported the child was outside the home or in another union council in all 12 SIAs. Among the total participants, 53 (16.3%) remained missed/could not be covered by the polio teams in any of the 12 SIAs (Table 3).

**Figure 2:**
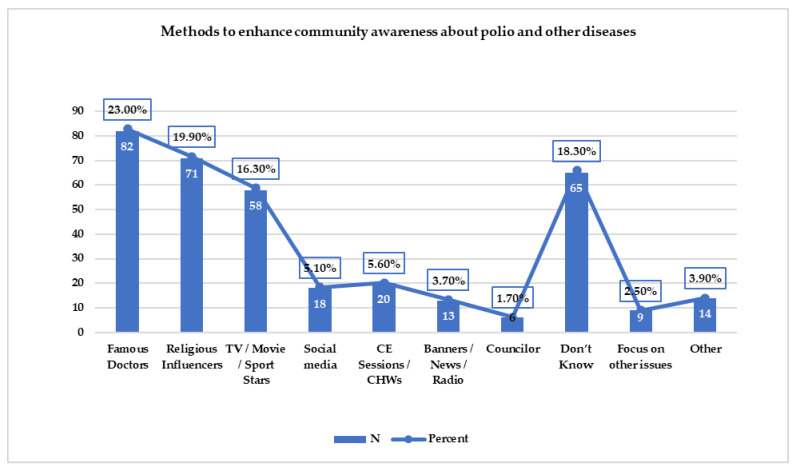
Suggestions from study participants on suitable ways to enhance community acceptance of polio vaccination campaigns in 34 high-risk union councils of Karachi division, Sindh, Pakistan.

Figure 2 displays the suggestions received from study participants detailing suitable ways to increase community acceptance of polio vaccination campaigns in 34 high-risk union councils of Karachi division, Sindh, Pakistan. The majority, 82 (23.0%), recommended a famous doctor, followed by 71 (19.9%) who recommended religious influencers, and 58 (16.3%) who recommended the involvement of sports and movie stars in community advocacy on the benefits of polio vaccination for children.

## 4. Discussion

Vaccination has always been opposed by communities, as is evident from a centuries-old literature review [14]. Therefore, exploring the reason for vaccine hesitancy helps the specific program gain insight into how to proceed in the future [15]. This is the first study that reports changes in the response of the same parent towards the polio campaigns over one year. We identified reasons for PMCs in polio vaccinations from parents of PMCs who were missed in three or more consecutive polio campaigns during the last year in high-risk UCs of Karachi. Every child who missed the polio vaccine endangers global eradication.

We also explored community awareness about polio disease and the means of awareness. We found that people who work in the polio program (i.e., front-line workers) were the main source of awareness regarding the polio campaigns and vaccination, followed by the media (television). A previous study also showed that social mobilization and community health worker visits improved awareness and injectable polio vaccine coverage in the community settings of Karachi, Pakistan [16]. Our study findings are contrary to those of a study by Facciolà et al., who reported that TV was the main source of the information regarding vaccination in the community [17]. We found that a major proportion of male children was persistently recorded missed during the last 12 SIAs. The probable reason for male baby refusal is the belief that OPV caused sterility in Pakistan. This finding is consistent with studies performed in the country in past [18].

It is evident from the available data that 0.1 million children in each national anti-polio campaign are missing OPV doses by refusing the vaccine. The major proportion of these refusals is from Pashtun sects which are influenced by the negative social media and the Peshawar incident. The refusals in Urdu-speaking pockets are also rising and are partly attributed to the adverse event that occurred during the typhoid conjugate vaccine (TCV) campaign in August 2021 in a district west of Karachi. The major portion of these refusals comes from slum areas of the province where there is a lack of routine immunization and WASH infrastructure. Therefore, there is a significant risk of persistent viral circulation in the province. In our study, approximately 81% of parents preferred private healthcare facilities for the treatment of childhood health problems, which also demonstrates their lack of trust in government-provided health facilities.

We found that among participants, half of the respondents reported that their children completed routine immunization vaccination courses, and a quarter also had EPI vaccination cards. This indicates that those refusing OPV also refuse essential immunizations. This is consistent with other studies and indicates that these individuals add to the circulation of wild poliovirus [19]. Every year a major bulk of new birth cohorts becomes vulnerable to poliomyelitis due to low routine immunization coverage in the country, as reported by previous studies [20].

Uniquely, we found that people believe that all OPV doses are not necessary. A very low percentage of respondents considered that the polio vaccine is highly important (full score 10) for the health of their children. Our finding contradicts the study performed in Pakistan in the year 2021 [6]. However, the population reported that polio teams visited their houses within a one-month period. The majority indicated that a polio team visited their houses in each campaign, irrespective of vaccine acceptance by the families. Despite the frequent visits from polio teams, a major portion of respondents reported that they did not know about polio infection. The fact that polio infection can cause paralysis was reported by 23% of households, and 40% of households reported that it is a type of disease. We also found that some households believed the monthly visits by the polio team were performed only to save their jobs or as per the requirement of the government. However, the literature search has documented that the polio team’s visit is inversely related to refusing the oral polio vaccine [21].

The major factor for refusing OPV was a lack of trust in vaccine quality, followed by fear of side effects, the household head not allowing polio vaccination, negative social media videos, and lack of trust in polio teams. Our findings on the negative use of social media for the polio vaccine were similar to those of a study in Charsada in 2020 [22]. Vaccine hesitancy is also affected by fear of injection, fear of side effects of the vaccine, and parents’ educational attainment, as reported in a cross-sectional study conducted in Indonesia [8]. A study conducted on parents’ vaccine hesitancy in Ireland also reported that parents had concerns about vaccine side effects, vaccine safety, and the number of vaccines administered [23].

Reasons for refusals were also documented in our study. We found that in 19% of cases, the reasons for missed children remained the same across 12 SIAs, whereas in 90% of cases, the reason for the missed child was changed by the parent of the child. The pattern of change in reasons for a missed child shows a switch in reasons of a missed child from one reason for refusal to another reason for refusal and a switch in reasons of refusal to not available and from not available to refusal in different campaigns. This pattern of reason change clearly shows hesitancy among parents toward polio vaccination. However, the same reasons, such as direct refusal followed by refusal due to some misconceptions and religious reasons, persisted in 12 SIAs. These findings are consistent with the studies performed in the country in the recent past [24]. We documented that vaccine hesitancy still remains high in the HRUCs. This emphasizes the need to enhance our understanding of the psychological needs of individuals in the 21st century, in which people are under pressure from various problems that impact their quality of life. In the current situation, the condition could have been caused by the COVID-19 pandemic also as the people have started to be very resistant to COVID-19 restrictions and thought that OPV is a Western conspiracy amid the COVID-19 situation as the population was experiencing lockdowns and mental health issues, as reported in a recent study in central and southern Italy by Barchielli et al. [25]. 

Moreover, the respondents also reported new strategies for increased vaccine acceptance in the community. Most study participants recommended that a famous doctor, religious influencer, or sports/movie star be engaged in the advocacy of polio vaccine uptake among children in high-risk areas of Karachi. These findings are novel. A study in Pakhtunkhwa reported that health journalists should be included as influencers for the acceptance of the oral polio vaccine [26].

This is also the first study conducted on parents of persistently missed children and in high-risk UCs which are the current focus of the polio program. Our study provides the base for re-structuring the community engagement mechanism, and importantly, our study was conducted in the high-risk UCs. However, despite these strengths, our reach was limited to 325 participants only. We could not collect a large sample size due to the increased security risk in the high-risk UCs after a major incident that occurred in the country. 

## 5. Conclusions

We concluded that misconception is still a big challenge, and the program needs to strive for community acceptance. The same children were recorded as missed multiple times in the 34 high-risk union councils of the Karachi division of Sindh province. Low levels of trust in vaccines and teams as well as fear of OPV side effects were among the main reason for vaccine hesitancy. Many parents were not aware that polio infection can cause life-long disability in children, and therefore they had little fear of the disease. The participant communities recommended involving famous medical doctors, religious influencers, and TV or sports stars to enhance knowledge and acceptance of polio vaccination.

## Figures and Tables

**Table 1:** Characteristics of study participants in 34 high-risk union councils of Karachi division, Sindh, Pakistan.

Variables	Number	Percentage
Districts		
Central	50	15.4
East	75	23.1
Korangi	10	3.1
Malir	40	12.3
South	10	3.1
West	140	43.0
Total children in the household		
Mean (±Standard Deviation)	3.391	2.428
Total under-five children in the household		
Mean (±Standard Deviation)	1.815	1.175
Gender of the child (under five)		
Female	141	43.4
Male	184	56.6
Common childhood health problems (multiple response variables)		
Respiratory tract infections	206	33
Gastrointestinal infections	123	19.7
Fever	205	32.8
Other infectious diseases	44	7.0
Other non-communicable diseases	33	5.3
No major health issues	14	2.2
When last visited health care facility for treatment of child		
Within the past 3 months	219	67.4
Between the last 3–6 months	47	14.5
Between 6–12 months	8	2.5
More than 12 months	8	2.5
Don’t know/don’t remember	43	13.1
Preferred facility for treatment of child		
Private	264	81.2
Government	28	8.7
Both	33	10.1
EPI routine vaccination status of children		
No	70	21.5
Yes (partial)	94	28.9
Yes (full)	161	49.6
EPI vaccination card availability		
No	186	57.2
Yes	105	32.3

**Table 2:** Knowledge, attitude, and perceptions of the community regarding polio vaccination in 34 high-risk union councils of Karachi division, Sindh, Pakistan.

Variables	Number	Percentage
Polio teams visit your house		
No	3	0.9
Yes	322	99.1
Number of times the Polio team visited		
1–10	40	12.3
11–24	194	59.7
≥36	80	24.6
Reason for repeated visits of polio teams (*multiple response variables*)		
Child health or vaccination	218	67.5
Job or Government Policy	115	35.6
conspiracy or data collection	45	13.9
To harm children	46	14.2
Don’t know	49	15.2
Rate behavior of the polio team (*0 very bad to 10 very good*)		
0	15	4.6
1–9	167	51.2
10	139	43.3
Participant’s knowledge of polio		
It’s a type of Paralysis	74	22.8
A type of a diseases	130	40
Don’t know	116	35.7
Participant’s knowledge about poliovirus-associated permanent disability		
No	79	24.3
Yes	241	74.2
Polio vaccine importance score (*0 not important to 10 highly important*)		
0	138	42.5
1–9	132	40.7
10	46	14.2
Ever refused for polio vaccine		
No	43	13.2
Yes	276	84.9
Reason for polio refusal (*multiple response variables*)		
no trust in vaccine quality	112	37.3
no trust in Polio Teams	20	6.7
afraid of side effects	45	15.0
Child was sick	31	10.3
Infertility	26	8.7
Misconception	30	10.0
Religious concerns	20	6.7
Elders don’t allow	42	14.0
Repeated Campaigns	13	4.3
Negative Video/Media	39	13.0
why this disease only	27	9.0
Prefer Private Vaccination	25	8.3
already vaccinated	2	0.7
Other reasons	14	4.7
No response	7	2.3
Anyone senior official came to convince for polio vaccination		
Don’t know	10	3.1
No	61	18.8
Yes	242	74.5
Vaccinated child as per someone’s recommendation (*multiple response variables*)		
No	136	45.5
CHW	33	11.0
Doctor	28	9.4
Other	102	33.9
The information source of the Polio campaign (*multiple response variables*)		
CHW	260	57.1
TV	68	14.9
Banner/pamphlet	49	10.8
Newspaper	15	3.3
Religious persons	8	1.8
Social Media/Mobile	8	1.8
Local Influencer/Neighbor/Relative	14	3.1
Other (Doctor, School)	33	7.3

**Table 3:** Status of persistently recorded missed children in 34 High-Risk Union Councils of Karachi division, Sindh, Pakistan.

Variables	Number	Percentage
Status of missed vaccination reasons		
Reason changed	263	80.9
Reason remained same	62	19.1
Persistent missed vaccination reasons		
Direct Refusal	9	2.8
Misconception	38	11.7
Religious Matter	1	0.3
Demands	2	0.6
Sickness	9	2.8
Outside UC	3	0.9
Reason changed	263	80.9
Vaccination coverage status in the last 12 SIAs		
Still missed child in all 12 SIAs	53	16.3
Once covered in the last 12 SIAs	75	23.1
Twice covered in the last 12 SIAs	49	15.1
Three or more times covered in the last 12 SIAs	148	45.5

## Data Availability

The datasets used and/or analyzed during the current study are available at the EOC Sindh (sindh.eoc@gmail.com) on reasonable request.

## Supplementary Materials

The following supporting information can be downloaded at: https://www.mdpi.com/article/10.3390/vaccines11010070/s1, Table S1: Union Council-wise distribution of study participants in 34 High-Risk Union Councils of Karachi division, Sindh, Pakistan. Table S2: Switch pattern in reasons of missed children from one reason of refusal to another reason for the refusal or not available in different campaigns from Jan 2018 to March 2019 SIAs in 34 High-Risk Union Councils of Karachi division, Sindh, Pakistan.
