# Supplementary material for: Vaccine Hesitancy and Perceptions of the Community about Polio in High-Risk Areas of Karachi, Sindh, Pakistan
Source: Vaccines (Basel). 2022 Dec 28;11(1):70. doi: 10.3390/vaccines11010070 (PMC9866813; doi:10.3390/vaccines11010070)
Supplement: Supplementary file 1 [file vaccines-11-00070-s001.zip › vaccines-2038022-supplementary.pdf]

vaccines

MDPI

Article

Vaccine Hesitancy and Perceptions of the Community About Polio in High-Risk Areas of Karachi, Sindh, Pakistan

Fayaz Hussain Abbasi [1], Ahmed Ali Shaikh [1,2], Jaishri Mehraj [1,3,*], Syed Musa Raza [1,4], Shumaila Rasool [1,5], Umar Farooq Bullo [1], Sandeep Mehraj [1,5], Zamir Ali Phul [1,5], Sundeep Sahitia [1,6], Asif Ali Zardari[1,6] and Shoukat Ali Chandio [1,4]

[1] Emergency Operations Centre for Polio Eradication and Immunization, Government of Sindh, Karachi, 75510, Pakistan
[2] Bill and Melinda Gates Foundation, Islamabad 44000, Pakistan
[3] Integral Global Health Inc., Islamabad 44000, Pakistan
[4] The United Nations Children's Fund (UNICEF), Islamabad 44050, Pakistan
[5] National Stop Transmission of Polio (N-STOP) Program, Karachi 75510, Pakistan
[6] World Health Organization (WHO), Islamabad 45500, Pakistan
* Correspondence: jaishrimehraj@gmail.com; Tel.: +00 92-21-35213035 (JM)

Table S1. Union Council-wise distribution of study participants in 34 High-Risk Union Councils of Karachi division, Sindh, Pakistan.

Union Councils	Number	Percent
BALOCH GOTH - 13	10	3.1
BANARAS COLONY - 7	10	3.1
BILAL COLONY - 1	10	3.1
***CHISTI NAGAR - 7**	10	3.1
CIVIL LINES - 9	10	3.1
FIRDOUS COLONY - 2	9	2.8
FRONTIER COLONY - 6	10	3.1
GARDEN EAST - 11	8	2.5
***GUJRO A**	10	3.1
***GUJRO B**	9	2.8
***GUJRO C**	10	3.1
***GUJRO D**	10	3.1
***GUJRO E**	10	3.1
GULZAR-E-HIJRAI - 12	2	0.6
ISLAM NAGAR - 3	10	3.1
***ISLAMIA COLONY - 9**	10	3.1
***ITTEHAD TOWN - 2**	10	3.1
KHAMISO GOTH - 9	10	3.1
MAMMARABAD – 6	10	3.1
***MANGOPIR – 8**	10	3.1
MOMINABAD – 1	10	3.1
***MUSLIMABAD – 2**	10	3.1
***MUZAFARABAD – 1**	10	3.1

NAZIMABAD -01 – 10	11	3.4
PAHAR GANJ – 2	10	3.1
PAPOSH NAGAR – 1	10	3.1
PEHALWAN GOTH – 10	10	3.1
QASBA COLONY – 8	10	3.1
QUAIDABAD – 4	10	3.1
REHRI – 2	10	3.1
SAFORA – 13	6	1.8
***SONGAL – 5**	10	3.1
SULTANABAD – 2	10	3.1
YOUSIF GOTH – 7	10	3.1
Total	325	100

* Super High-Risk Union Councils of Karachi.

Table S2. Switch pattern in reasons of missed children from one reason of refusal to another reason for the refusal or not available in different campaigns from Jan 2018 to March 2019 SIAs in 34 High-Risk Union Councils of Karachi division, Sindh, Pakistan.

S. No.	ID number	Jan-18	Feb-18	Mar-18	Apr-18	May-18	Jul-18	Aug-18	Sep-18	Nov-18	Dec-18	Jan-19	Mar-19	Persistent Missed Reason
1	K15	Direct Refusal	Direct Refusal	Direct Refusal	Direct Refusal	Direct Refusal	Direct Refusal	Direct Refusal	Direct Refusal	Direct Refusal	Direct Refusal	Direct Refusal	Direct Refusal	**Direct Refusal**
2	OR58	Direct Refusal	Direct Refusal	Direct Refusal	Direct Refusal	Direct Refusal	Direct Refusal	Direct Refusal	Direct Refusal	Direct Refusal	Direct Refusal	Direct Refusal	Direct Refusal	**Direct Refusal**
3	OR34	Direct Refusal	Direct Refusal	Direct Refusal	Direct Refusal	Direct Refusal	Direct Refusal	Direct Refusal	Direct Refusal	Direct Refusal	Direct Refusal	Direct Refusal	Direct Refusal	**Direct Refusal**
4	LQT20	Direct Refusal	Direct Refusal	Direct Refusal	Direct Refusal	Direct Refusal	Direct Refusal	Direct Refusal	Direct Refusal		Direct Refusal	Direct Refusal	Direct Refusal	**Direct Refusal**
5	OR10	Direct Refusal		Direct Refusal	Direct Refusal	Direct Refusal	Direct Refusal	Direct Refusal	Direct Refusal	Direct Refusal	Direct Refusal		Direct Refusal	**Direct Refusal**
6	OR34	Direct Refusal	Direct Refusal	Direct Refusal	Direct Refusal	Direct Refusal	Direct Refusal	Direct Refusal	Direct Refusal	Direct Refusal	Direct Refusal	Direct Refusal	Direct Refusal	**Direct Refusal**
7	SD11	Direct Refusal	Direct Refusal		Direct Refusal			Direct Refusal	Direct Refusal	Direct Refusal	Direct Refusal	Direct Refusal	Direct Refusal	**Direct Refusal**
8	SI42	Direct Refusal	Direct Refusal	Direct Refusal	Direct Refusal	Direct Refusal	Direct Refusal	Direct Refusal	Direct Refusal	Direct Refusal		Direct Refusal	Direct Refusal	**Direct Refusal**
9	G71									Direct Refusal	Direct Refusal	Direct Refusal	Direct Refusal	**Direct Refusal**
10	B38	Misconception	Misconception	Misconception	Misconception	Misconception	Misconception	Misconception	Misconception	Misconception	Misconception	Misconception	Misconception	**Misconception**

**11**	**NA10**	Misconception		Misconception	Misconception	Misconception	Misconception	Misconception	Misconception	Misconception	Misconception	Misconception	Misconception	**Misconception**
**12**	**OR65**	Misconception	Misconception	Misconception	Misconception	Misconception	Misconception	Misconception	Misconception	Misconception	Misconception	Misconception	Misconception	**Misconception**
**13**	**B39**	Misconception	Misconception	Misconception	Misconception	Misconception	Misconception	Misconception	Misconception	Misconception	Misconception	Misconception	Misconception	**Misconception**
**14**	**LQT31**	Misconception	Misconception		Misconception	Misconception	Misconception	Misconception	Misconception	Misconception	Misconception	Misconception	Misconception	**Misconception**
**15**	**SI20**	Misconception	Misconception	Misconception	Misconception	Misconception	Misconception	Misconception	Misconception	Misconception	Misconception	Misconception	Misconception	**Misconception**
**16**	**BQ29**	Misconception	Misconception	Misconception	Misconception	Misconception	Misconception	Misconception	Misconception	Misconception	Misconception	Misconception	Misconception	**Misconception**
**17**	**SI29**	Misconception	Misconception	Misconception	Misconception	Misconception	Misconception	Misconception	Misconception	Misconception	Misconception	Misconception	Misconception	**Misconception**
**18**	**OR54**	Misconception	Misconception	Misconception	Misconception	Misconception	Misconception	Misconception	Misconception	Misconception	Misconception		Misconception	**Misconception**
**19**	**OR55**	Misconception	Misconception	Misconception	Misconception	Misconception	Misconception	Misconception	Misconception	Misconception	Misconception		Misconception	**Misconception**
**20**	**OR71**	Misconception	Misconception	Misconception	Misconception	Misconception	Misconception	Misconception	Misconception	Misconception		Misconception	Misconception	**Misconception**
**21**	**OR61**	Misconception		Misconception	Misconception	Misconception	Misconception	Misconception	Misconception	Misconception		Misconception	Misconception	**Misconception**
**22**	**K18**	Misconception	Misconception	Misconception	Misconception	Misconception	Misconception	Misconception	Misconception	Misconception	Misconception	Misconception	Misconception	**Misconception**
**23**	**GI2**	Misconception	Misconception	Misconception	Misconception	Misconception	Misconception	Misconception	Misconception	Misconception	Misconception	Misconception	Misconception	**Misconception**
**24**	**NOR3**		Misconception	Misconception	Misconception	Misconception	Misconception	Misconception	Misconception	Misconception	Misconception	Misconception	Misconception	**Misconception**
**25**	**K14**	Misconception	Misconception	Misconception	Misconception	Misconception	Misconception	Misconception	Misconception	Misconception	Misconception	Misconception	Misconception	**Misconception**
**26**	**J6**	Misconception	Misconception	Misconception	Misconception	Misconception	Misconception	Misconception	Misconception	Misconception	Misconception	Misconception	Misconception	**Misconception**
**27**	**OR62**	Misconception	Misconception	Misconception	Misconception	Misconception	Misconception	Misconception		Misconception	Misconception	Misconception	Misconception	**Misconception**
**28**	**G168**					Misconception	Misconception	Misconception			Misconception	Misconception	Misconception	**Misconception**
**29**	**BQ9**	Misconception	Misconception	Misconception	Misconception	Misconception	Misconception	Misconception	Misconception	Misconception	Misconception	Misconception	Misconception	**Misconception**
**30**	**SI55**	Misconception	Misconception	Misconception	Misconception	Misconception	Misconception	Misconception	Misconception	Misconception	Misconception	Misconception	Misconception	**Misconception**
**31**	**NA25**	Misconception		Misconception	Misconception	Misconception	Misconception	Misconception	Misconception	Misconception	Misconception	Misconception	Misconception	**Misconception**

**32**	**G74**									Misconception	Misconception	Misconception	Misconception	**Misconception**
**33**	**SI10**		Misconception	Misconception	Misconception	Misconception	Misconception	Misconception	Misconception	Misconception	Misconception	Misconception	Misconception	**Misconception**
**34**	**BQ11**	Misconception	Misconception	Misconception	Misconception	Misconception	Misconception	Misconception	Misconception	Misconception	Misconception	Misconception	Misconception	**Misconception**
**35**	**GI23**	Misconception	Misconception	Misconception	Misconception	Misconception	Misconception	Misconception	Misconception	Misconception	Misconception	Misconception	Misconception	**Misconception**
**36**	**LQT36**	Misconception	Misconception		Misconception	Misconception	Misconception	Misconception	Misconception	Misconception	Misconception	Misconception	Misconception	**Misconception**
**37**	**SI28**	Misconception			Misconception	Misconception	Misconception	Misconception	Misconception	Misconception	Misconception	Misconception	Misconception	**Misconception**
**38**	**SI16**	Misconception	Misconception	Misconception	Misconception	Misconception	Misconception	Misconception	Misconception	Misconception	Misconception	Misconception	Misconception	**Misconception**
**39**	**BQ8**	Misconception	Misconception	Misconception	Misconception	Misconception	Misconception	Misconception	Misconception	Misconception		Misconception	Misconception	**Misconception**
**40**	**BQ12**	Misconception	Misconception	Misconception	Misconception	Misconception	Misconception	Misconception	Misconception	Misconception	Misconception	Misconception	Misconception	**Misconception**
**41**	**NA18**	Misconception	Misconception	Misconception	Misconception	Misconception	Misconception	Misconception	Misconception	Misconception	Misconception	Misconception	Misconception	**Misconception**
**42**	**SI18**	Misconception	Misconception	Misconception	Misconception	Misconception	Misconception	Misconception	Misconception	Misconception	Misconception	Misconception	Misconception	**Misconception**
**43**	**BQ20**	Misconception	Misconception	Misconception	Misconception	Misconception	Misconception	Misconception	Misconception	Misconception	Misconception	Misconception	Misconception	**Misconception**
**44**	**B43**	Misconception	Misconception	Misconception	Misconception	Misconception	Misconception	Misconception	Misconception	Misconception	Misconception	Misconception	Misconception	**Misconception**
**45**	**BQ19**	Misconception	Misconception	Misconception	Misconception	Misconception	Misconception	Misconception	Misconception	Misconception	Misconception	Misconception	Misconception	**Misconception**
**46**	**BQ14**	Misconception	Misconception	Misconception	Misconception	Misconception	Misconception	Misconception	Misconception	Misconception	Misconception	Misconception	Misconception	**Misconception**
**47**	**BQ26**	Misconception	Misconception	Misconception	Misconception	Misconception	Misconception	Misconception	Misconception	Misconception	Misconception	Misconception	Misconception	**Misconception**
**48**	**BQ13**	Religious Matter	Religious Matter	Religious Matter	Religious Matter	Religious Matter	Religious Matter	Religious Matter	Religious Matter	Religious Matter	Religious Matter	Religious Matter	Religious Matter	**Religious Matter**
**49**	**K9**	Demands	Demands	Demands	Demands	Demands	Demands	Demands	Demands	Demands	Demands	Demands	Demands	**Demands**
**50**	**B45**	Demands	Demands	Demands	Demands	Demands	Demands	Demands	Demands	Demands	Demands	Demands	Demands	**Demands**
**51**	**LAN14**	Sickness	Sickness	Sickness	Sickness	Sickness	Sickness	Sickness	Sickness	Sickness	Sickness	Sickness	Sickness	**Sickness**
**52**	**KA7**	Sickness	Sickness	Sickness	Sickness	Sickness	Sickness	Sickness	Sickness	Sickness	Sickness	Sickness	Sickness	**Sickness**
**53**	**G18**	Sickness	Sickness		Sickness	Sickness	Sickness	Sickness	Sickness		Sickness	Sickness	Sickness	**Sickness**

**54**	**G160**	Sickness	Sickness	Sickness	Sickness	Sickness	Sickness	Sickness	Sickness	Sickness	Sickness	Sickness	Sickness	**Sickness**
**55**	**SI15**	Sickness	Sickness	Sickness	Sickness	Sickness	Sickness	Sickness		Sickness	Sickness	Sickness		**Sickness**
**56**	**K20**	Sickness	Sickness	Sickness	Sickness	Sickness	Sickness	Sickness	Sickness	Sickness	Sickness	Sickness	Sickness	**Sickness**
**57**	**G16**	Sickness	Sickness	Sickness	Sickness	Sickness	Sickness	Sickness	Sickness	Sickness	Sickness	Sickness	Sickness	**Sickness**
**58**	**G78**	Sickness		Sickness	Sickness	Sickness	Sickness	Sickness	Sickness	Sickness	Sickness	Sickness	Sickness	**Sickness**
**59**	**SD12**	Sickness	Sickness	Sickness	Sickness	Sickness	Sickness	Sickness	Sickness	Sickness		Sickness	Sickness	**Sickness**
**60**	**G84**	Outside UC								Outside UC		Outside UC	Outside UC	**Outside UC**
**61**	**NOR9**		Outside UC	Outside UC	Outside UC	Outside UC	Outside UC	Outside UC	Outside UC	Outside UC	Outside UC	Outside UC	Outside UC	**Outside UC**
**62**	**G83**				Outside UC			Outside UC	Outside UC	Outside UC			Outside UC	**Outside UC**
**63**	**BQ1**	Locked House	Misconception	Misconception	Misconception	Misconception	Misconception	Misconception	Misconception	Misconception	Misconception	Misconception	Misconception	**Reason change**
**64**	**OR11**	Misconception		Misconception	Misconception	Misconception	Misconception		Misconception	Misconception	Misconception	Locked House	Misconception	**Reason change**
**65**	**G146**	Misconception			Misconception	Misconception	Misconception	Misconception	Locked House	Misconception	Misconception	Misconception	Misconception	**Misconception**
**66**	**G3**	Outside UC	Outside UC	Outside UC	Outside UC		Misconception	Outside UC	Outside UC	Outside UC	Outside UC	Outside UC	Outside UC	**Reason change**
**67**	**OR26**	Direct Refusal	Direct Refusal	Direct Refusal	Direct Refusal	Direct Refusal	Misconception	Direct Refusal		Locked House	Locked House	Locked House		**Reason change**
**68**	**SI23**	Direct Refusal		Locked House	Sickness	Locked House	Direct Refusal	Direct Refusal	Misconception	Direct Refusal	Direct Refusal	Direct Refusal	Direct Refusal	**Reason change**
**69**	**LAN28**		Misconception	Misconception	Misconception	Misconception	Repeated Campaigns		Repeated Campaigns	Outside UC	Direct Refusal	Misconception	Misconception	**Reason change**
**70**	**KA19**	Misconception	Outside UC	Misconception	Misconception	Misconception	Sickness	Misconception	Repeated Campaigns	Direct Refusal	Direct Refusal	Direct Refusal	Misconception	**Reason change**
**71**	**G134**	Outside UC	Direct Refusal	Outside UC		Direct Refusal		Misconception	Misconception	Misconception	Misconception	Misconception	Misconception	**Reason change**
**72**	**OR28**	Misconception	Inside UC	Misconception	Misconception	Misconception	Misconception	Misconception	Repeated Campaigns	Direct Refusal	Direct Refusal	Direct Refusal	Direct Refusal	**Reason change**
**73**	**LAN6**	Direct Refusal	Direct Refusal	Direct Refusal	Direct Refusal	Misconception	Direct Refusal	Outside UC	Misconception	Misconception	Misconception	Misconception	Outside UC	**Reason change**
**74**	**OR48**	Direct Refusal	Direct Refusal	Outside UC	Direct Refusal	Misconception	Misconception	Misconception	Misconception	Misconception	Misconception	Misconception	Misconception	**Reason change**
**75**	**LAN3**	Misconception	Religious Matter	Religious Matter	Direct Refusal	Religious Matter	Direct Refusal	Direct Refusal	Religious Matter	Religious Matter	Religious Matter	Religious Matter	Outside UC	**Reason change**

76	**OR12**	Misconception	Outside UC	Misconception	Misconception	Outside UC	Misconception	Misconception	Misconception	Misconception	Misconception	Misconception	Outside UC	**Reason change**
77	**LQT3**	Misconception	Outside UC	Misconception	Misconception		Religious Matter	Misconception	Misconception	Misconception	Misconception	Locked House	Misconception	**Reason change**
78	**LAN11**	Direct Refusal	Direct Refusal	Direct Refusal	Direct Refusal	Direct Refusal	Direct Refusal		Direct Refusal	Religious Matter	Outside UC	Religious Matter	Religious Matter	**Reason change**
79	**G69**	Inside UC	Direct Refusal	Direct Refusal	Direct Refusal	Direct Refusal	Direct Refusal	Direct Refusal	Direct Refusal	Direct Refusal	Misconception		Misconception	**Reason change**
80	**SD16**	Sickness	Sickness	Sickness	Sickness	Sickness	Sickness	Sickness		Direct Refusal	Outside UC	Sickness		**Reason change**
81	**LQT23**		Direct Refusal		Outside UC	Misconception	Direct Refusal	Misconception	Misconception	Outside UC	Misconception	Misconception	Misconception	**Reason change**
82	**G140**	Outside UC	Outside UC	Direct Refusal	Direct Refusal	Direct Refusal	Misconception	Misconception	Direct Refusal	Misconception	Misconception	Misconception	Misconception	**Reason change**
83	**G103**	Outside UC	Direct Refusal		Direct Refusal		Outside UC	Direct Refusal	Direct Refusal	Outside UC	Repeated Campaigns	Misconception	Misconception	**Reason change**
84	**G111**	Direct Refusal	Direct Refusal		Inside UC		Outside UC	Outside UC	Outside UC	Outside UC	Direct Refusal	Inside UC	Outside UC	**Reason change**
85	**G135**			Direct Refusal	Demands	Demands	Demands	Demands	Demands	Outside UC	Misconception	Outside UC	Outside UC	**Reason change**
86	**LQT9**	Demands	Inside UC	Direct Refusal	Sickness	Direct Refusal	Direct Refusal	Direct Refusal	Direct Refusal	Direct Refusal	Direct Refusal	Direct Refusal	Direct Refusal	**Reason change**
87	**KA3**	Outside UC	Direct Refusal	Direct Refusal	Direct Refusal	Misconception	Misconception	Misconception	Misconception	Misconception	Misconception	Misconception	Misconception	**Reason change**
88	**LQT12**	Inside UC		Misconception	Demands	In school	Misconception	Misconception	Misconception	Misconception	Misconception	Misconception	Misconception	**Reason change**
89	**G40**		Inside UC	Repeated Campaigns	Repeated Campaigns	Repeated Campaigns	Direct Refusal	Direct Refusal	Repeated Campaigns	Locked House	Outside UC	Misconception	Misconception	**Reason change**
90	**G36**	Direct Refusal	Sickness	Misconception	Direct Refusal	Direct Refusal	Direct Refusal	Misconception	Sickness		Direct Refusal	Outside UC	Outside UC	**Reason change**
91	**G153**	Outside UC	Misconception	Misconception	Direct Refusal		Direct Refusal	Misconception	Misconception	Misconception	Misconception	Misconception	Misconception	**Reason change**
92	**G118**	Direct Refusal	Direct Refusal		Inside UC		Outside UC	Outside UC	Outside UC	Outside UC	Direct Refusal	Inside UC	Outside UC	**Reason change**
93	**LQT18**			Outside UC	Outside UC	Outside UC	Outside UC	Outside UC	Direct Refusal	Direct Refusal	Direct Refusal	Direct Refusal	Misconception	**Reason change**
94	**LQT7**	Direct Refusal	Outside UC	Direct Refusal	Inside UC	Outside UC			Inside UC	Inside UC		Inside UC	Outside UC	**Reason change**
95	**G127**			Outside UC	Outside UC	Religious Matter	Outside UC	Outside UC	Direct Refusal	Misconception	Misconception	Misconception	Misconception	**Reason change**

**96**	**SD1**	Direct Refusal	Direct Refusal	Direct Refusal	Repeated Campaigns	Direct Refusal	Direct Refusal	Direct Refusal	Direct Refusal	Direct Refusal	Direct Refusal	Direct Refusal	Outside UC	**Reason change**
**97**	**LAN9**	Direct Refusal	Religious Matter	Religious Matter	Religious Matter	Religious Matter	Religious Matter	Religious Matter	Direct Refusal	Outside UC	Misconception	Sickness	Religious Matter	**Reason change**
**98**	**NA19**	Religious Matter	Religious Matter	Religious Matter	Religious Matter	Religious Matter			Outside UC	Misconception	Outside UC	Misconception	Misconception	**Reason change**
**99**	**OR4**	Direct Refusal		Direct Refusal	Direct Refusal	Outside UC	Outside UC	Direct Refusal	Misconception	Misconception		Misconception	Misconception	**Reason change**
**100**	**G117**	Direct Refusal	Sickness		Inside UC	Direct Refusal	Direct Refusal	Direct Refusal	Direct Refusal	Direct Refusal	Direct Refusal	Direct Refusal	Direct Refusal	**Reason change**
**101**	**LAN29**	Religious Matter	Misconception	Misconception		Misconception	Misconception		Outside UC	Locked House	Direct Refusal	Misconception	Demands	**Reason change**
**102**	**OR22**	Locked House	Locked House		Direct Refusal	Misconception	Misconception	Misconception	Misconception	Misconception	Misconception	Sickness	Sickness	**Reason change**
**103**	**G150**		Outside UC	Misconception	Misconception	Misconception	Direct Refusal	Misconception	Misconception		Outside UC		Misconception	**Reason change**
**104**	**LAN30**	Religious Matter		Misconception		Misconception	Misconception		Outside UC	Locked House	Direct Refusal	Misconception	Demands	**Reason change**
**105**	**G8**	Outside UC		Misconception	Direct Refusal	Misconception		Misconception	Misconception				Outside UC	**Reason change**
**106**	**G176**		Outside UC		Outside UC		Outside UC	Outside UC	Outside UC	Misconception	Inside UC		Sickness	**Reason change**
**107**	**G87**						Direct Refusal	Direct Refusal	Direct Refusal	Outside UC	Misconception	Misconception	Misconception	**Reason change**
**108**	**OR37**	Inside UC	Repeated Campaigns	Inside UC	Misconception	Misconception	Misconception	Direct Refusal	Direct Refusal	Direct Refusal	Direct Refusal	Direct Refusal	Direct Refusal	**Reason change**
**109**	**OR36**	Inside UC	Religious Matter	Religious Matter	Locked House	Misconception	Misconception	Direct Refusal	Direct Refusal	Direct Refusal	Outside UC	Direct Refusal	Direct Refusal	**Reason change**
**110**	**Nor12**		Locked House	Locked House	Locked House	Inside UC	Inside UC	Direct Refusal	Sickness	Direct Refusal	Direct Refusal	Direct Refusal	Direct Refusal	**Reason change**
**111**	**G79**	Misconception	Misconception	Misconception						Misconception	Inside UC	Misconception	Misconception	**Reason change**
**112**	**SI24**	Direct Refusal		Locked House	Sickness	Locked House	Direct Refusal	Direct Refusal	Misconception	Direct Refusal	Direct Refusal	Direct Refusal	Direct Refusal	**Reason change**
**113**	**SI25**		Direct Refusal	Direct Refusal	Misconception	Misconception	Misconception	Misconception	Misconception	Misconception	Outside UC	Misconception	Misconception	**Reason change**
**114**	**LQT1**		Direct Refusal		Direct Refusal	Direct Refusal	Direct Refusal	Direct Refusal	Direct Refusal				Outside UC	**Reason change**
**115**	**G161**				Direct Refusal	Outside UC	Direct Refusal		Misconception			Sickness	Outside UC	**Reason change**

**116**	**G49**									Direct Refusal	Outside UC	Direct Refusal	Direct Refusal	**Reason change**
**117**	**KA2**	Sickness	Outside UC	Sickness	Sickness	Sickness	Sickness	Sickness	Sickness	Sickness	Sickness	Sickness	Sickness	**Reason change**
**118**	**G175**	Direct Refusal		Outside UC	Direct Refusal	Misconception	Misconception	Direct Refusal	Direct Refusal	Direct Refusal	Direct Refusal	Misconception	Direct Refusal	**Reason change**
**119**	**G169**		Demands	Misconception	Misconception		Misconception	Direct Refusal	Outside UC	Misconception	Misconception	Misconception	Direct Refusal	**Reason change**
**120**	**G159**			Direct Refusal	Direct Refusal	Direct Refusal	Direct Refusal	Outside UC	Locked House	Outside UC	Direct Refusal	Misconception	Misconception	**Reason change**
**121**	**G157**			Outside UC	Locked House	Inside UC	Locked House	Direct Refusal	Direct Refusal	Direct Refusal	Direct Refusal	Outside UC	Inside UC	**Reason change**
**122**	**G62**		Locked House	Locked House					Sickness	Locked House		Locked House	Locked House	**Reason change**
**123**	**SI30**	Misconception	Misconception			Misconception	Misconception	Misconception	Misconception	Outside UC	Misconception	Misconception	Misconception	**Reason change**
**124**	**OR45**	Direct Refusal	Direct Refusal	Outside UC	Direct Refusal	Locked House	Locked House	Locked House		Direct Refusal	Demands	Direct Refusal		**Reason change**
**125**	**LAN7**	Misconception		Misconception	Repeated Campaigns	Outside UC	Outside UC	Direct Refusal	Direct Refusal	Misconception	Misconception	Misconception	Misconception	**Reason change**
**126**	**GI51**	Direct Refusal	Outside UC	Misconception	Direct Refusal		Direct Refusal	Direct Refusal	Direct Refusal	Direct Refusal	Outside UC		Outside UC	**Reason change**
**127**	**NA20**	Misconception	Misconception	Repeated Campaigns	Misconception	Misconception	Misconception	Misconception		Misconception	Misconception		Outside UC	**Reason change**
**128**	**NA26**	Outside UC	Outside UC	Direct Refusal	Direct Refusal	Direct Refusal	Direct Refusal	Direct Refusal	Direct Refusal	Direct Refusal	Misconception	Misconception	Misconception	**Reason change**
**129**	**SI57**	Sickness	Sickness	Sickness	Sickness	Outside UC	Misconception	Sickness	Direct Refusal	Sickness	Sickness	Sickness	Sickness	**Reason change**
**130**	**G131**	Outside UC	Outside UC	Direct Refusal	Outside UC	Outside UC	Outside UC	Direct Refusal	Misconception	Misconception	Direct Refusal	Demands	Sickness	**Reason change**
**131**	**G152**	Outside UC	Demands	Demands	Misconception	Misconception	Misconception	Misconception	Misconception	Misconception	Misconception	Misconception	Misconception	**Reason change**
**132**	**NOR2**		Sickness	Outside UC	Inside UC	Direct Refusal	Outside UC	Direct Refusal	Misconception	Misconception	Misconception	Misconception	Inside UC	**Reason change**
**133**	**G5**	Misconception	Religious Matter	Religious Matter	Religious Matter	Religious Matter	Misconception	Misconception	Religious Matter				Outside UC	**Reason change**
**134**	**G37**	Repeated Campaigns			Direct Refusal	Outside UC	Direct Refusal	Misconception	Direct Refusal	Inside UC	Repeated Campaigns	Misconception	Misconception	**Reason change**

135	G13	Sickness		Misconception	Misconception	Misconception	Outside UC		Sickness			Locked House	Sickness	Reason change
136	G59	Inside UC	Outside UC						Inside UC	Outside UC	Outside UC		Direct Refusal	Reason change
137	G58	Inside UC	Outside UC						Inside UC	Outside UC	Outside UC		Direct Refusal	Reason change
138	G52						Outside UC		Outside UC	Outside UC		Outside UC	Direct Refusal	Reason change
139	SI58	Locked House	Locked House	Locked House	Locked House	Direct Refusal	Direct Refusal	Direct Refusal	Locked House	Direct Refusal	Religious Matter	Religious Matter	Religious Matter	Reason change
140	SI52	Religious Matter	Inside UC	Direct Refusal	Religious Matter	Direct Refusal	Misconception	Direct Refusal	Misconception	Misconception	Misconception	Misconception	Misconception	Reason change
141	J9	Direct Refusal	Direct Refusal	Misconception	Misconception	Misconception	Misconception	Misconception	Misconception	Misconception	Misconception	Outside UC	Misconception	Reason change
142	LQT35	Direct Refusal	Misconception		Misconception	Misconception	Misconception	Outside UC	Religious Matter	Misconception	Sickness	Repeated Campaigns	Repeated Campaigns	Reason change
143	G33	Misconception	Misconception	Misconception	Religious Matter	Misconception	Outside UC	Misconception	Misconception	Misconception	Misconception	Misconception	Misconception	Reason change
144	J11	Outside UC		Outside UC	Misconception	Misconception	Misconception	Misconception	Misconception	Misconception	Misconception	Misconception	Misconception	Reason change
145	G130		Outside UC		Outside UC	Outside UC	Inside UC	Outside UC	Outside UC	Inside UC	Direct Refusal	Sickness	Direct Refusal	Reason change
146	LAN32			Direct Refusal	Direct Refusal	Direct Refusal	Direct Refusal	Outside UC	Outside UC	Direct Refusal	Misconception	Misconception	Repeated Campaigns	Reason change
147	G134	Outside UC	Direct Refusal	Outside UC		Direct Refusal		Misconception	Misconception	Misconception	Misconception	Misconception	Misconception	Reason change
148	G80	Direct Refusal	Direct Refusal	Direct Refusal	Direct Refusal	Direct Refusal	Direct Refusal	Direct Refusal	Outside UC	Outside UC			Outside UC	Reason change
149	Nor5			Inside UC	Direct Refusal	Direct Refusal	Direct Refusal		Misconception	Misconception	Direct Refusal	Misconception	Misconception	Reason change
150	G9	Sickness	Sickness	Sickness		Misconception		Locked House	Outside UC				Misconception	Reason change
151	LAN33	Misconception	Misconception	Outside UC	Misconception	Outside UC	Misconception	Misconception	Direct Refusal	Direct Refusal	Direct Refusal	Misconception	Misconception	Reason change
152	SI54	Inside UC	Locked House	Locked House	Locked House	Repeated Campaigns	Locked House	Direct Refusal	Misconception	Misconception	Misconception	Misconception	Misconception	Reason change
153	B46	Misconception	Misconception	Misconception	Misconception	Misconception	Misconception	Misconception	Outside UC	Direct Refusal	Direct Refusal	Misconception	Misconception	Reason change

**154**	**G171**	Locked House			Religious Matter	Religious Matter	Outside UC	Misconception	Religious Matter	Religious Matter	Direct Refusal	Direct Refusal	Direct Refusal	**Reason change**
**155**	**G104**	Direct Refusal			Direct Refusal	Direct Refusal	Direct Refusal	Direct Refusal	Direct Refusal	Misconception	Demands		Outside UC	**Reason change**
**156**	**G156**			Direct Refusal	Direct Refusal	Direct Refusal	Direct Refusal	Locked House	Direct Refusal	Demands	Demands	Direct Refusal	Demands	**Reason change**
**157**	**G162**	Outside UC				Misconception	Misconception	Misconception	Misconception	Direct Refusal	Direct Refusal	Direct Refusal	Direct Refusal	**Reason change**
**158**	**G173**	Outside UC		Misconception	Inside UC	Misconception	Direct Refusal	Direct Refusal	Direct Refusal	Sickness		Sickness	Sickness	**Reason change**
**159**	**KA13**	Sickness	Locked House		Sickness	Repeated Campaigns		Sickness	Misconception	Misconception	Misconception		Misconception	**Reason change**
**160**	**B33**	Sickness	Inside UC	Misconception	Misconception	Misconception	Sickness	Misconception	Direct Refusal	Direct Refusal	Direct Refusal	Direct Refusal	Misconception	**Reason change**
**161**	**G12**	Misconception		Direct Refusal	Direct Refusal	Direct Refusal	Locked House	Misconception	Repeated Campaigns	Direct Refusal	Direct Refusal	Sickness	Sickness	**Reason change**
**162**	**LAN42**	Misconception	Misconception	Misconception	Misconception	Misconception	Misconception	Locked House		Direct Refusal	Misconception	Misconception	Sickness	**Reason change**
**163**	**G97**		Direct Refusal	Locked House	Direct Refusal	Direct Refusal				Misconception	Direct Refusal	Direct Refusal	Misconception	**Reason change**
**164**	**SI60**	Outside UC		Sickness	Religious Matter	Direct Refusal	Direct Refusal	Direct Refusal	Misconception	Misconception	Misconception	Misconception	Misconception	**Reason change**
**165**	**SI66**	Direct Refusal	Direct Refusal	Direct Refusal	Direct Refusal	Direct Refusal	Locked House	Direct Refusal	Direct Refusal	Misconception	Misconception	Misconception	Misconception	**Reason change**
**166**	**G10**	Direct Refusal	Direct Refusal	Direct Refusal	Locked House	Direct Refusal	Direct Refusal	Direct Refusal	Direct Refusal	Direct Refusal	Direct Refusal	Direct Refusal	Direct Refusal	**Reason change**
**167**	**GI28**	Misconception	Direct Refusal	Misconception	Direct Refusal	Direct Refusal	Direct Refusal	Direct Refusal	Misconception	Misconception	Misconception	Misconception	Locked House	**Reason change**
**168**	**GI7**	Misconception	Misconception	Repeated Campaigns	Misconception	Misconception		Misconception	Misconception	Misconception	Misconception	Misconception	Misconception	**Reason change**
**169**	**LAN10**	Direct Refusal	Direct Refusal	Direct Refusal	Direct Refusal	Direct Refusal	Direct Refusal	Direct Refusal	Direct Refusal	Religious Matter	Misconception	Misconception	Religious Matter	**Reason change**
**170**	**SD7**	Direct Refusal	Direct Refusal	Direct Refusal	Sickness	Direct Refusal	Direct Refusal	Direct Refusal	Direct Refusal	Direct Refusal	Direct Refusal	Direct Refusal	Religious Matter	**Reason change**
**171**	**SI1**	Sickness	Direct Refusal	Direct Refusal	Misconception	Misconception	Misconception	Misconception	Misconception	Misconception	Direct Refusal	Misconception	Misconception	**Reason change**
**172**	**BQ36**	Misconception	Misconception	Misconception	Misconception	Misconception	Misconception	Misconception	Misconception	Misconception	Misconception	Repeated Campaigns	Misconception	**Reason change**

**173**	**B42**	Direct Refusal	Direct Refusal	Direct Refusal	Direct Refusal	Direct Refusal	Direct Refusal	Direct Refusal	Direct Refusal	Direct Refusal	Direct Refusal	Misconception	Misconception	**Reason change**
**174**	**K3**	Direct Refusal	Direct Refusal	Direct Refusal	Direct Refusal	Direct Refusal	Direct Refusal	Direct Refusal	Direct Refusal	Direct Refusal	Direct Refusal	Direct Refusal	Misconception	**Reason change**
**175**	**SI6**	Misconception	Misconception	Misconception	Misconception	Direct Refusal	Direct Refusal	Direct Refusal	Sickness	Sickness	Direct Refusal	Direct Refusal	Direct Refusal	**Reason change**
**176**	**GI69**	Direct Refusal	Direct Refusal	Direct Refusal	Direct Refusal	Misconception	Direct Refusal	Direct Refusal	Direct Refusal	Misconception	Misconception	Misconception	Misconception	**Reason change**
**177**	**B26**	Sickness	Sickness	Sickness	Sickness	Sickness	Sickness	Sickness	Sickness	Sickness	Sickness	Sickness	Misconception	**Reason change**
**178**	**B40**	Misconception	Misconception	Misconception	Misconception	Direct Refusal	Direct Refusal	Direct Refusal	Misconception	Misconception	Misconception	Religious Matter	Misconception	**Reason change**
**179**	**G115**	Direct Refusal	Direct Refusal	Direct Refusal	Direct Refusal	Direct Refusal	Direct Refusal	Demands	Direct Refusal	Direct Refusal	Direct Refusal	Direct Refusal	Direct Refusal	**Reason change**
**180**	**KA15**	Direct Refusal	Direct Refusal	Direct Refusal	Sickness	Direct Refusal	Demands	Direct Refusal	Direct Refusal	Direct Refusal	Direct Refusal	Direct Refusal	Direct Refusal	**Reason change**
**181**	**OR50**	Misconception	Sickness	Misconception	Misconception	Sickness	Sickness	Sickness	Misconception	Misconception	Misconception	Misconception	Misconception	**Reason change**
**182**	**NOR7**	Misconception	Sickness	Sickness	Sickness	Sickness	Sickness	Sickness	Sickness	Sickness	Sickness	Sickness	Sickness	**Reason change**
**183**	**NA4**	Religious Matter	Religious Matter		Direct Refusal	Direct Refusal	Direct Refusal	Direct Refusal	Direct Refusal	Direct Refusal	Direct Refusal	Direct Refusal	Direct Refusal	**Reason change**
**184**	**LQT27**	Direct Refusal	Repeated Campaigns		Direct Refusal	Direct Refusal	Misconception	Misconception	Misconception	Misconception	Misconception	Misconception	Repeated Campaigns	**Reason change**
**185**	**SI3**	Direct Refusal	Direct Refusal	Direct Refusal	Direct Refusal	Direct Refusal	Direct Refusal	Direct Refusal	Direct Refusal	Direct Refusal	Direct Refusal	Misconception	Misconception	**Reason change**
**186**	**OR44**	Repeated Campaigns	Repeated Campaigns	Repeated Campaigns	Repeated Campaigns	Repeated Campaigns	Repeated Campaigns	Repeated Campaigns		Direct Refusal	Direct Refusal	Direct Refusal		**Reason change**
**187**	**LAN12**	Direct Refusal	Direct Refusal	Direct Refusal	Direct Refusal	Direct Refusal	Direct Refusal	Direct Refusal	Direct Refusal	Religious Matter	Misconception	Religious Matter	Religious Matter	**Reason change**
**188**	**OR43**	Sickness	Sickness	Sickness	Sickness	Sickness	Sickness	Sickness		Misconception	Misconception	Sickness		**Reason change**
**189**	**G138**	Direct Refusal		Direct Refusal	Direct Refusal	Direct Refusal	Direct Refusal	Religious Matter	Misconception	Religious Matter	Religious Matter	Religious Matter	Religious Matter	**Reason change**
**190**	**K1**	Demands	Misconception	Misconception	Misconception	Misconception	Misconception	Misconception	Misconception	Misconception	Misconception	Misconception	Misconception	**Reason change**
**191**	**GI30**	Misconception	Misconception	Misconception	Misconception	Misconception	Misconception	Misconception	Misconception	Repeated Campaigns	Misconception	Misconception	Misconception	**Reason change**

**192**	**GI12**	Misconception	Misconception	Misconception	Misconception	Misconception	Misconception	Misconception	Misconception	Direct Refusal	Misconception	Misconception	Misconception	**Reason change**
**193**	**OR7**	Direct Refusal	Direct Refusal	Direct Refusal	Direct Refusal	Direct Refusal	Direct Refusal	Direct Refusal	Misconception	Misconception	Misconception	Misconception	Misconception	**Reason change**
**194**	**B19**	Demands	Demands	Demands	Repeated Campaigns	Demands	Demands	Demands	Direct Refusal	Demands	Demands	Demands	Misconception	**Reason change**
**195**	**Nor13**	Sickness	Sickness	Sickness	Sickness	Misconception	Sickness	Sickness	Sickness	Sickness	Sickness	Sickness	Sickness	**Reason change**
**196**	**LQT33**	Repeated Campaigns	Misconception	Misconception	Repeated Campaigns		Misconception	Misconception	Misconception	Misconception	Misconception	Misconception	Misconception	**Reason change**
**197**	**OR29**	Misconception	Misconception	Misconception	Misconception	Misconception	Direct Refusal		Direct Refusal	Misconception	Misconception	Misconception	Misconception	**Reason change**
**198**	**OR49**	Misconception	Misconception	Misconception	Demands	Misconception	Misconception		Misconception	Misconception	Misconception		Misconception	**Reason change**
**199**	**B20**	Direct Refusal	Direct Refusal	Direct Refusal	Direct Refusal	Direct Refusal	Direct Refusal	Direct Refusal	Direct Refusal		Direct Refusal		Misconception	**Reason change**
**200**	**SI47**	Misconception	Misconception	Misconception	Misconception	Misconception	Misconception	Misconception		Religious Matter	Misconception	Misconception		**Reason change**
**201**	**NA12**	Direct Refusal	Direct Refusal		Direct Refusal	Direct Refusal	Direct Refusal	Direct Refusal	Direct Refusal	Misconception	Misconception	Misconception	Misconception	**Reason change**
**202**	**Nor15**	Sickness	Direct Refusal	Direct Refusal	Direct Refusal	Direct Refusal	Direct Refusal	Direct Refusal		Direct Refusal	Direct Refusal	Misconception		**Reason change**
**203**	**SI8**	Direct Refusal	Direct Refusal	Direct Refusal	Direct Refusal	Direct Refusal	Direct Refusal	Direct Refusal	Direct Refusal	Demands			Misconception	**Reason change**
**204**	**SI45**	Direct Refusal	Direct Refusal	Direct Refusal	Direct Refusal	Direct Refusal	Direct Refusal	Direct Refusal	Direct Refusal	Religious Matter	Direct Refusal		Direct Refusal	**Reason change**
**205**	**NA13**	Direct Refusal	Direct Refusal		Misconception	Misconception	Misconception	Misconception	Misconception	Misconception	Misconception	Misconception	Misconception	**Reason change**
**206**	**OR41**	Misconception	Direct Refusal	Direct Refusal	Direct Refusal	Direct Refusal	Direct Refusal	Direct Refusal	Direct Refusal	Direct Refusal	Direct Refusal	Direct Refusal	Direct Refusal	**Reason change**
**207**	**LAN31**	Misconception	Misconception	Misconception		Misconception		Sickness	Misconception	Direct Refusal	Religious Matter	Repeated Campaigns	Misconception	**Reason change**
**208**	**OR6**			Direct Refusal	Misconception	Direct Refusal	Direct Refusal	Direct Refusal	Misconception	Misconception	Misconception		Misconception	**Reason change**
**209**	**G143**	Demands	Direct Refusal	Direct Refusal	Direct Refusal	Direct Refusal	Direct Refusal	Direct Refusal	Direct Refusal	Direct Refusal	Direct Refusal	Direct Refusal	Direct Refusal	**Reason change**
**210**	**LQT5**	Sickness				Sickness	Sickness	Direct Refusal			Direct Refusal	Direct Refusal	Locked House	**Reason change**

**211**	**G88**						Direct Refusal	Direct Refusal	Misconception	Direct Refusal	Direct Refusal	Direct Refusal	Misconception	**Reason change**
**212**	**G100**						Misconception	Direct Refusal	Direct Refusal	Direct Refusal	Misconception		Religious Matter	**Reason change**
**213**	**G90**							Direct Refusal	Misconception	Direct Refusal	Direct Refusal		Direct Refusal	**Reason change**
**214**	**G46**								Direct Refusal	Direct Refusal		Demands	Sickness	**Reason change**
**215**	**BQ31**	Sickness	Sickness	Misconception	Misconception	Misconception	Misconception	Misconception	Misconception	Misconception	Misconception	Misconception	Misconception	**Reason change**
**216**	**GI1**	Direct Refusal	Misconception	Misconception	Misconception	Misconception	Misconception	Misconception	Misconception	Misconception	Misconception	Misconception	Misconception	**Reason change**
**217**	**GI27**	Direct Refusal	Misconception	Misconception	Misconception	Misconception	Misconception	Misconception	Misconception	Misconception	Misconception	Misconception	Misconception	**Reason change**
**218**	**B6**	Direct Refusal	Direct Refusal	Direct Refusal	Direct Refusal	Direct Refusal	Direct Refusal	Direct Refusal	Direct Refusal	Direct Refusal	Direct Refusal	Repeated Campaigns	Misconception	**Reason change**
**219**	**SI69**	Misconception	Misconception	Direct Refusal	Misconception	Misconception	Misconception	Misconception		Religious Matter	Direct Refusal	Direct Refusal		**Reason change**
**220**	**KA9**	Misconception	Direct Refusal	Direct Refusal	Direct Refusal	Repeated Campaigns	Direct Refusal	Demands	Sickness	Direct Refusal			Direct Refusal	**Reason change**
**221**	**G77**	Repeated Campaigns	Direct Refusal	Direct Refusal	Direct Refusal		Direct Refusal	Direct Refusal	Misconception	Misconception	Misconception	Misconception	Direct Refusal	**Reason change**
**222**	**KA14**		Misconception	Misconception	Misconception	Misconception	Misconception	Misconception	Misconception	Misconception	Repeated Campaigns	Misconception	Misconception	**Reason change**
**223**	**G163**			Misconception	Direct Refusal		Direct Refusal	Sickness	Direct Refusal	Sickness	Sickness	Sickness	Sickness	**Reason change**
**224**	**G56**				Misconception	Misconception	Misconception		Misconception	Misconception	Misconception	Direct Refusal	Direct Refusal	**Reason change**
**225**	**GI5**	Misconception	Misconception	Misconception	Misconception	Misconception	Misconception	Direct Refusal	Misconception	Misconception	Misconception	Misconception	Misconception	**Reason change**
**226**	**NA22**	Misconception	Misconception	Misconception	Misconception	Misconception	Direct Refusal	Direct Refusal	Misconception	Misconception	Misconception	Misconception	Misconception	**Reason change**
**227**	**G70**	Direct Refusal	Direct Refusal	Repeated Campaigns	Misconception	Direct Refusal	Direct Refusal	Direct Refusal	Direct Refusal	Direct Refusal	Direct Refusal		Direct Refusal	**Reason change**
**228**	**LQT24**	Direct Refusal	Direct Refusal		Misconception	Misconception	Misconception	Misconception	Direct Refusal	Direct Refusal	Misconception	Direct Refusal	Direct Refusal	**Reason change**

229	K7	Misconception	Direct Refusal	Misconception	Misconception	Misconception	Misconception	Misconception	Misconception			Sickness	Misconception	**Reason change**
230	nor10	Direct Refusal	Direct Refusal	Direct Refusal	Direct Refusal		Direct Refusal	Direct Refusal	Direct Refusal		Direct Refusal	Misconception	Misconception	**Reason change**
231	G93	Direct Refusal		Misconception	Misconception	Misconception	Misconception	Misconception	Misconception	Direct Refusal			Direct Refusal	**Reason change**
232	J3	Direct Refusal	Misconception	Misconception	Misconception	Misconception	Misconception	Misconception	Religious Matter	Misconception	Misconception	Misconception	Misconception	**Reason change**
233	LQT29	Direct Refusal	Direct Refusal		Direct Refusal	Direct Refusal	Direct Refusal	Direct Refusal	Misconception	Misconception	Misconception	Religious Matter	Religious Matter	**Reason change**
234	LQT26	Direct Refusal	Repeated Campaigns		Direct Refusal	Direct Refusal	Misconception	Misconception	Misconception	Misconception	Misconception	Misconception	Repeated Campaigns	**Reason change**
235	K13	Misconception	Misconception	Misconception	Misconception	Religious Matter	Misconception	Misconception	Misconception	Misconception	Misconception	Misconception	Misconception	**Reason change**
236	G31	Misconception	Repeated Campaigns	Misconception	Repeated Campaigns	Direct Refusal	Repeated Campaigns	Repeated Campaigns	Repeated Campaigns	Direct Refusal	Repeated Campaigns	Direct Refusal	Direct Refusal	**Reason change**
237	OR25	Direct Refusal	Direct Refusal	Direct Refusal	Direct Refusal	Direct Refusal	Direct Refusal	Direct Refusal		Misconception	Misconception	Misconception		**Reason change**
238	SI2		Direct Refusal	Direct Refusal	Misconception	Misconception	Misconception	Misconception	Misconception	Misconception	Direct Refusal	Misconception	Misconception	**Reason change**
239	G172	Repeated Campaigns			Religious Matter	Religious Matter	Religious Matter	Religious Matter	Religious Matter	Sickness	Religious Matter	Religious Matter	Direct Refusal	**Reason change**
240	G57				Misconception	Misconception	Misconception	Misconception	Misconception	Misconception	Misconception	Direct Refusal	Direct Refusal	**Reason change**
241	GI14	Misconception	Misconception	Misconception	Misconception	Misconception	Repeated Campaigns	Misconception	Misconception	Repeated Campaigns	Misconception	Misconception	Misconception	**Reason change**
242	BQ32	Misconception	Misconception	Misconception	Misconception	Misconception	Misconception	Misconception	Misconception	Misconception	Misconception	Misconception	Repeated Campaigns	**Reason change**
243	SI26	Misconception	Direct Refusal	Misconception	Misconception	Misconception	Misconception	Misconception	Misconception	Misconception	Misconception	Misconception	Misconception	**Reason change**
244	J14	Direct Refusal	Direct Refusal	Misconception	Misconception	Misconception	Misconception	Misconception	Misconception	Misconception	Misconception	Misconception	Misconception	**Reason change**
245	KA5	Misconception	Misconception	Direct Refusal	Misconception	Direct Refusal	Misconception	Misconception	Misconception	Misconception	Misconception	Misconception	Misconception	**Reason change**
246	G128	Direct Refusal	Sickness	Direct Refusal	Direct Refusal	Direct Refusal	Sickness	Direct Refusal	Sickness	Direct Refusal	Direct Refusal	Direct Refusal	Direct Refusal	**Reason change**

247	SI12	Misconception	Misconception	Misconception	Misconception	Misconception	Misconception	Demands	Direct Refusal	Direct Refusal	Direct Refusal	Misconception	Misconception	**Reason change**
248	GI50	Misconception	Misconception	Misconception	Misconception	Misconception	Misconception	Misconception	Misconception	Misconception	Direct Refusal	Direct Refusal	Misconception	**Reason change**
249	G147	Misconception	Misconception	Misconception	Misconception	Demands	Misconception	Misconception	Misconception	Misconception	Misconception		Misconception	**Reason change**
250	K5	Misconception	Misconception	Misconception	Misconception		Misconception	Misconception	Direct Refusal	Misconception	Misconception	Misconception	Misconception	**Reason change**
251	NA28	Misconception	Misconception	Misconception	Misconception	Misconception	Misconception	Misconception	Religious Matter	Misconception	Misconception	Misconception	Misconception	**Reason change**
252	NA14	Direct Refusal	Direct Refusal		Direct Refusal	Direct Refusal	Direct Refusal	Direct Refusal	Demands	Direct Refusal	Direct Refusal	Direct Refusal	Direct Refusal	**Reason change**
253	GI57	Misconception	Misconception	Direct Refusal	Direct Refusal	Sickness	Direct Refusal	Direct Refusal	Direct Refusal	Direct Refusal	Misconception		Misconception	**Reason change**
254	NA1	Direct Refusal	Direct Refusal		Direct Refusal	Direct Refusal	Direct Refusal	Direct Refusal	Demands	Misconception	Misconception	Misconception	Misconception	**Reason change**
255	BQ2	Misconception	Sickness	Sickness	Sickness	Sickness	Sickness	Sickness	Sickness	Misconception	Sickness	Sickness	Misconception	**Reason change**
256	NA5	Misconception	Misconception	Misconception	Direct Refusal	Direct Refusal	Misconception		Misconception	Misconception	Misconception	Misconception	Misconception	**Reason change**
257	J13	Direct Refusal	Direct Refusal	Misconception	Misconception	Misconception	Misconception	Misconception	Misconception	Misconception	Misconception		Misconception	**Reason change**
258	G27	Misconception	Direct Refusal	Sickness	Sickness	Sickness	Sickness	Sickness	Sickness		Direct Refusal	Sickness	Sickness	**Reason change**
259	SI68	Direct Refusal	Sickness	Direct Refusal	Misconception	Misconception	Misconception	Misconception	Misconception	Direct Refusal	Direct Refusal	Direct Refusal	Direct Refusal	**Reason change**
260	NA31	Direct Refusal	Direct Refusal	Direct Refusal	Direct Refusal	Direct Refusal	Direct Refusal	Direct Refusal		Direct Refusal	Misconception	Misconception		**Reason change**
261	NA15	Demands	Direct Refusal	Direct Refusal	Direct Refusal	Direct Refusal	Direct Refusal	Direct Refusal		Direct Refusal	Demands	Direct Refusal		**Reason change**
262	SI9	Religious Matter	Religious Matter		Misconception	Misconception	Misconception	Misconception	Religious Matter	Religious Matter	Religious Matter	Religious Matter	Misconception	**Reason change**
263	NA11	Direct Refusal	Direct Refusal		Direct Refusal	Direct Refusal	Direct Refusal	Direct Refusal	Direct Refusal	Misconception	Religious Matter	Religious Matter	Religious Matter	**Reason change**
264	OR24	Direct Refusal	Direct Refusal	Direct Refusal	Direct Refusal	Direct Refusal	Direct Refusal	Direct Refusal		Misconception	Misconception	Misconception		**Reason change**
265	SI51	Sickness	Sickness	Sickness		Sickness	Direct Refusal	Sickness	Sickness	Misconception		Misconception	Misconception	**Reason change**
266	B8	Sickness	Sickness	Sickness	Sickness	Sickness	Sickness		Direct Refusal	Direct Refusal	Direct Refusal	Misconception	Misconception	**Reason change**

**267**	**NA29**	Misconception	Repeated Campaigns	Misconception	Misconception	Misconception	Misconception	Misconception	Misconception	Misconception	Misconception	Misconception	Misconception	**Reason change**
**268**	**G138**	Direct Refusal		Direct Refusal	Direct Refusal	Direct Refusal	Direct Refusal	Religious Matter	Misconception	Religious Matter	Religious Matter	Religious Matter	Religious Matter	**Reason change**
**269**	**NOR8**		Direct Refusal	Direct Refusal	Demands	Demands	Direct Refusal		Repeated Campaigns	Misconception	Repeated Campaigns	Misconception	Misconception	**Reason change**
**270**	**SD13**	Direct Refusal		Direct Refusal	Repeated Campaigns		Direct Refusal	Direct Refusal	Direct Refusal	Misconception	Misconception	Misconception	Misconception	**Reason change**
**271**	**G123**		Direct Refusal	Direct Refusal	Direct Refusal	Direct Refusal	Direct Refusal	Demands	Direct Refusal	Direct Refusal	Direct Refusal	Direct Refusal	Direct Refusal	**Reason change**
**272**	**G35**	Religious Matter	Direct Refusal	Direct Refusal	Direct Refusal	Direct Refusal	Direct Refusal	Direct Refusal	Misconception		Misconception	Misconception	Misconception	**Reason change**
**273**	**G144**			Direct Refusal	Direct Refusal	Locked House	Direct Refusal	Direct Refusal	Demands	Misconception	Demands	Misconception	Misconception	**Reason change**
**274**	**G86**						Direct Refusal		Repeated Campaigns	Misconception	Direct Refusal	Direct Refusal	Direct Refusal	**Reason change**
**275**	**NA2**	Religious Matter	Religious Matter	Religious Matter	Religious Matter	Religious Matter	Religious Matter	Religious Matter	Misconception	Religious Matter	Religious Matter	Religious Matter	Misconception	**Reason change**
**276**	**KA8**	Sickness	Sickness	Direct Refusal	Sickness	Direct Refusal	Demands	Direct Refusal	Sickness	Sickness	Sickness	Sickness	Sickness	**Reason change**
**277**	**B37**	Demands	Demands	Demands	Demands	Demands	Demands	Demands	Demands	Misconception	Misconception	Misconception	Misconception	**Reason change**
**278**	**BQ15**	Religious Matter	Religious Matter	Religious Matter	Religious Matter	Religious Matter	Religious Matter	Religious Matter	Religious Matter	Religious Matter	Misconception	Religious Matter	Misconception	**Reason change**
**279**	**SD3**	Direct Refusal	Sickness	Sickness	Sickness	Sickness	Sickness	Sickness	Sickness	Sickness	Sickness	Sickness	Sickness	**Reason change**
**280**	**SI5**	Sickness	Sickness	Sickness	Sickness	Sickness	Sickness	Direct Refusal	Direct Refusal	Sickness	Sickness	Sickness	Sickness	**Reason change**
**281**	**GI36**	Misconception	Misconception	Misconception	Misconception	Misconception	Misconception	Misconception	Misconception	Misconception	Misconception	Direct Refusal	Religious Matter	**Reason change**
**282**	**LAN21**	Misconception	Direct Refusal	Misconception	Misconception	Misconception	Misconception	Misconception		Misconception	Misconception	Misconception	Misconception	**Reason change**
**283**	**LQT11**		Misconception	Misconception	Misconception	Misconception	Religious Matter	Misconception	Misconception	Misconception	Misconception	Misconception	Religious Matter	**Reason change**
**284**	**LQT29**	Direct Refusal	Direct Refusal		Direct Refusal	Direct Refusal	Direct Refusal	Direct Refusal	Misconception	Misconception	Misconception	Religious Matter	Religious Matter	**Reason change**

285	LQT34	Direct Refusal	Direct Refusal	Direct Refusal		Direct Refusal	Direct Refusal	Repeated Campaigns	Misconception	Misconception	Misconception	Misconception	Misconception	Reason change
286	BQ27	Misconception	Misconception	Misconception	Misconception	Misconception	Misconception	Misconception	Misconception	Misconception	Repeated Campaigns	Repeated Campaigns	Misconception	Reason change
287	SI43	Misconception		Religious Matter	Religious Matter	Religious Matter	Misconception	Religious Matter	Misconception	Misconception	Direct Refusal	Religious Matter	Misconception	Reason change
288	SI44	Religious Matter	Religious Matter	Religious Matter	Religious Matter		Religious Matter	Religious Matter	Religious Matter	Religious Matter	Direct Refusal	Religious Matter	Religious Matter	Reason change
289	SI41	Direct Refusal	Direct Refusal	Religious Matter	Misconception		Religious Matter	Misconception	Religious Matter	Religious Matter	Religious Matter	Misconception	Misconception	Reason change
290	GI58	Direct Refusal	Direct Refusal	Direct Refusal	Direct Refusal		Demands	Direct Refusal	Direct Refusal	Direct Refusal	Direct Refusal	Misconception	Misconception	Reason change
291	SI36	Religious Matter	Religious Matter	Religious Matter	Repeated Campaigns		Misconception	Demands	Direct Refusal	Misconception	Misconception	Misconception	Religious Matter	Reason change
292	B2	Direct Refusal	Direct Refusal	Direct Refusal	Direct Refusal		Direct Refusal	Misconception	Misconception	Direct Refusal	Direct Refusal	Misconception	Misconception	Reason change
293	BQ30	Misconception	Misconception		Misconception	Misconception	Misconception	Religious Matter	Repeated Campaigns	Misconception	Misconception	Misconception	Misconception	Reason change
294	LAN24	Direct Refusal	Repeated Campaigns	Misconception	Misconception	Misconception		Misconception	Misconception	Misconception	Sickness	Repeated Campaigns	Misconception	Reason change
295	GI52	Direct Refusal	Direct Refusal	Direct Refusal	Direct Refusal	Direct Refusal	Direct Refusal	Direct Refusal	Direct Refusal	Misconception	Misconception	Misconception	Misconception	Reason change
296	BQ34	Direct Refusal	Direct Refusal	Misconception	Misconception	Misconception	Misconception	Misconception	Misconception	Misconception	Misconception	Misconception	Misconception	Reason change
297	NA23	Misconception			Misconception	Misconception	Misconception	Misconception	Religious Matter	Misconception	Religious Matter	Misconception	Misconception	Reason change
298	G120	Misconception	Misconception	Misconception	Misconception	Misconception	Misconception	Misconception	Misconception	Misconception	Repeated Campaigns	Misconception	Sickness	Reason change
299	G34	Repeated Campaigns	Repeated Campaigns	Misconception	Repeated Campaigns	Misconception		Direct Refusal	Sickness	Direct Refusal	Direct Refusal	Direct Refusal	Direct Refusal	Reason change
300	LAN36	Misconception	Misconception		Religious Matter	Misconception	Misconception	Misconception	Religious Matter	Direct Refusal	Direct Refusal	Misconception	Direct Refusal	Reason change
301	B12					Sickness	Direct Refusal	Direct Refusal	Direct Refusal	Direct Refusal	Direct Refusal	Misconception	Misconception	Reason change

**302**	**SD15**	Direct Refusal		Direct Refusal	Direct Refusal	Direct Refusal	Direct Refusal	Direct Refusal	Direct Refusal	Direct Refusal	Misconception	Misconception	Misconception	**Reason change**
**303**	**G155**		Sickness		Misconception	Misconception	Misconception	Misconception	Misconception	Misconception	Misconception	Misconception	Misconception	**Reason change**
**304**	**G92**	Sickness	Repeated Campaigns	Sickness	Sickness					Direct Refusal	Direct Refusal	Direct Refusal	Direct Refusal	**Reason change**
**305**	**G81**			Repeated Campaigns	Direct Refusal	Direct Refusal	Misconception	Direct Refusal	Direct Refusal	Direct Refusal	Misconception	Direct Refusal	Misconception	**Reason change**
**306**	**G4**	Direct Refusal	Direct Refusal	Misconception	Misconception	Repeated Campaigns			Misconception	Misconception		Misconception	Misconception	**Reason change**
**307**	**G39**	Repeated Campaigns				Direct Refusal	Repeated Campaigns	Repeated Campaigns	Repeated Campaigns	Repeated Campaigns	Direct Refusal	Religious Matter	Religious Matter	**Reason change**
**308**	**G85**					Direct Refusal				Direct Refusal	Direct Refusal	Misconception	Misconception	**Reason change**
**309**	**LAN44**	Direct Refusal	Direct Refusal	Direct Refusal	Direct Refusal	Sickness	Misconception	Misconception	Misconception	Misconception	Misconception	Misconception	Misconception	**Reason change**
**310**	**LAN8**	Direct Refusal	Direct Refusal	Misconception	Misconception	Direct Refusal	Direct Refusal	Direct Refusal	Direct Refusal	Misconception	Misconception	Religious Matter	Misconception	**Reason change**
**311**	**B41**	Direct Refusal	Direct Refusal	Direct Refusal	Direct Refusal	Direct Refusal	Direct Refusal	Misconception	Direct Refusal	Direct Refusal	Direct Refusal	Direct Refusal	Direct Refusal	**Reason change**
**312**	**BQ25**	Sickness	Misconception	Misconception	Misconception	Misconception	Misconception	Misconception	Misconception	Misconception	Misconception	Misconception	Misconception	**Reason change**
**313**	**B10**	Direct Refusal	Direct Refusal	Misconception	Misconception	Direct Refusal	Religious Matter	Misconception	Misconception	Misconception	Misconception	Misconception	Misconception	**Reason change**
**314**	**SI34**	Direct Refusal	Direct Refusal	Sickness	Direct Refusal	Direct Refusal	Direct Refusal	Direct Refusal	Direct Refusal	Direct Refusal	Direct Refusal	Direct Refusal	Direct Refusal	**Reason change**
**315**	**J12**	Religious Matter	Misconception	Misconception	Misconception	Misconception	Misconception	Misconception	Misconception	Misconception	Misconception	Misconception	Misconception	**Reason change**
**316**	**SI39**	Religious Matter	Religious Matter	Direct Refusal	Misconception	Misconception	Religious Matter	Religious Matter	Religious Matter	Religious Matter	Religious Matter	Religious Matter	Religious Matter	**Reason change**
**317**	**B13**	Sickness	Direct Refusal	Direct Refusal	Direct Refusal	Direct Refusal	Direct Refusal	Direct Refusal	Direct Refusal	Direct Refusal	Direct Refusal	Misconception	Misconception	**Reason change**
**318**	**GI43**	Misconception	Misconception	Misconception	Misconception	Misconception	Misconception	Misconception	Misconception	Misconception	Direct Refusal	Misconception	Misconception	**Reason change**
**319**	**SI32**	Direct Refusal	Direct Refusal	Direct Refusal	Direct Refusal	Direct Refusal	Direct Refusal	Direct Refusal	Direct Refusal	Direct Refusal	Direct Refusal	Direct Refusal	Misconception	**Reason change**
**320**	**B30**	Direct Refusal	Misconception	Demands	Direct Refusal	Direct Refusal	Direct Refusal	Direct Refusal	Demands	Direct Refusal	Direct Refusal	Misconception	Misconception	**Reason change**

**321**	**J19**	Direct Refusal	Direct Refusal	Religious Matter	Religious Matter	Religious Matter	Religious Matter	Religious Matter	Religious Matter	Religious Matter	Religious Matter	Religious Matter	Religious Matter	**Reason change**
**322**	**SD10**	Locked House	Locked House	Locked House	Locked House	Locked House	Locked House	Locked House	Locked House	Locked House		Outside UC	Locked House	**Reason change**
**323**	**SD2**	Outside UC	Outside UC	Outside UC	Outside UC	Outside UC	Outside UC	Outside UC	Locked House	Inside UC	Outside UC	Locked House	Outside UC	**Reason change**
**324**	**G66**						Locked House	Outside UC	Locked House	Outside UC		Outside UC	Outside UC	**Reason change**
**325**	**G50**									Outside UC	Inside UC	Outside UC	Outside UC	**Reason change**

Supplementary material: Cronbach's Score for the reliability of the questions_

RELIABILITY OF QUESTIONNAIRE

DATE OF TESTING:

PLACE OF TESTING: RHC Kemari

SAMPLE SIZE: 10 Respondents were selected randomly from the Outpatient department of the Rural Health Center.

DATA COLLECTOR: Staff trained on the questionnaire and principal investigator

DATA COLLECTION METHOD: Face-to-face interview

METHODS:

A total of 10 interviews were conducted to calculate Cronbach's Score for the reliability of the questions. The data was cleaned and put in excel for analysis. Two variances were calculated each for the individual question and the total variance of the questionnaire. Of all 25 questions, only questions no 4 and 6 were needing more elaboration by the data collector as requested by the respondent. However, remaining all questions were answered in a single go. The total time to complete each questionnaire was 5 to 7 minutes. Cronbach's coefficient was used to test the reliability of the questionnaire. We got Cronbach's Coefficient of 0.70 which shows the content of the questionnaire was acceptable. All questions were culturally acceptable to the community.

Formula:

$$\alpha = \frac{n}{n-1}\left(1 - \frac{\sum s^2 (X_i)}{s^2(Y)}\right)$$

Bachelor Print

Taken from Website; https://www.bachelorprint.com/statistics/cronbachs-alpha/

**Test-Retest Validity of Questionnaire**	
**#items/questions**	**25**
**Sum of item variance**	**4.19**
**The variance of total score**	**15.43**
**Cronbach's Alpha Coefficient**	**0.70**

Conclusion:

The questionnaire assessed in this study proved to be a valid and reliable tool to measure the outcome of interest. Since it is easy to understand and can be completed by the participants in a short time.

Financial support and sponsorship

Nil

Conflicts of interest

There are no conflicts of interest

THANK YOU

Supplementary material: Master trainer's performance indicators

Performance Indicators – Lead Trainers

S. No.	KPIs (60% weightage)	Definition	S. No.	Attributes / Soft Skills (30% weightage)	Definition
1	**Coordination**	▪ DPCR<br>▪ EOC	1	**Communication and Interpersonal Skills**	Effective communication with the district (DPCR and Provincial EOC)
2	**Training Planning**	▪ Ensures the training is supported by TNA<br>▪ Agenda development and necessary Modification<br>▪ Review of training material/ methodologies<br>▪ Logistic planning of sessions	2	**Leadership**	▪ Stakeholder management<br>▪ Confidence<br>▪ Motivates/assists his team<br>▪ Mental Maturity – knows how to behave in different situations<br>▪ Empowers the team<br>▪ Mentoring and coaching
3	**Field Support**	▪ Venue mapping and validation<br>▪ Supportive supervision in the field	3	**Organized Approach**	▪ Is able to manage various tasks in an efficient and timely manner<br>▪ Resource management
4	**Supervision**	▪ Attendance<br>▪ Field deployment planning<br>▪ Ensuring quality training implementation	4	**Professional Conduct**	▪ Maintains a composed demeanor<br>▪ Wears culture-appropriate clothes<br>▪ Talks respectfully<br>▪ Show ethnic and cultural sensitivity
5	**Reporting**	▪ Weekly work plan<br>▪ Compiled feedback report after each phase of training<br>▪ Daily reports<br>▪ Monthly reports<br>▪ Quarterly Training reports	5	**Result Orientation**	▪ Is mindful of programmatic goals<br>▪ Achieves results on timely basis
